# Assessment of the effect of serum and follicular fluid vitamin D and glucose on assisted reproductive technique outcome: A cross-sectional study

**DOI:** 10.18502/ijrm.v20i3.10714

**Published:** 2022-04-21

**Authors:** Robabe Hosseinisadat, Lida Saeed, Anis Ghasemirad, Victoria Habibzadeh, Sedigheh Safar Heidari

**Affiliations:** ^1^Department of Obstetrics and Gynecology, School of Medicine, Kerman University of Medical Sciences, Kerman, Iran.; ^2^Afzalipour Infertility Center, Kerman University of Medical Sciences, Kerman, Iran.

**Keywords:** Vitamin D, Glucose, Follicular fluid, Pregnancy, Assisted reproductive techniques.

## Abstract

**Background:**

Vitamin D and glucose play an important role in the female reproductive system.

**Objective:**

The aim of this study was to assess the effect of serum and follicular fluid vitamin D on assisted reproductive technique (ART) outcomes.

**Materials and Methods:**

102 infertile women were enrolled in the study. All cases received the routine in vitro fertilization protocol. On the oocyte retrieval day, a sample of their peripheral blood and follicular fluid was obtained to determine the level of vitamin D and glucose. We also evaluated ART outcomes including oocytes, 2 pronucleus and embryo number, implantation, chemical and clinical pregnancy, and abortion rate. Finally, the effect of serum and follicular fluid vitamin D and glucose on the ART outcomes was assessed.

**Results:**

There was no difference in the characteristics, serum vitamin D, follicular fluid vitamin D, fasting blood sugar (FBS), or follicular fluid glucose between the women with vs. without a positive clinical pregnancy. There was no significant difference between the ART outcomes based on vitamin D level. The mean follicular fluid glucose levels in women who were deficient, insufficient and sufficient in vitamin D were 65.20 ± 14.65, 63.47 ± 14.90 and 55.97 ± 15.64, respectively. Follicular fluid glucose was lower in women with sufficient vitamin D levels and this difference was statistically significant (p = 0.01). There was no relationship between the three follicular fluid vitamin D levels and ART outcomes. In women with normal FBS levels, the level of follicular fluid vitamin D was significantly lower than in the women with pre-diabetic FBS status (p < 0.001).

**Conclusion:**

The present study showed that serum vitamin D level, follicular fluid vitamin D level, FBS, and follicular fluid sugar were not predictive parameters for ART outcomes.

## 1. Introduction

Oocyte maturation depends on cytoplasmic and nuclear changes, and each defect in this process can reduce the chances of successful fertilization (1). Some studies have shown that a lack of many vitamins and nutrients can reduce the chances of successful natural fertility, and have confirmed the effect of vitamins and supplements on embryo development (2, 3). Follicular fluid is an important medium for the development of oocytes. Increasing or decreasing the structure of this fluid has negative effects on the oocyte and embryo morphology and quality (4).

Vitamin D is a fat-soluble steroid hormone which is essential for the production of sex hormones, estradiol and progesterone (5). Vitamin D connects to its receptor and then activates the molecular pathways in cells or tissues (6). There is a vitamin D receptor in the human ovaries, uterus, endometrium and placenta. Experiments have shown that vitamin D can support endometrium decidualization and regulatory factors for the expression of some genes such as Homeobox genes 10, and that it is important for embryo implantation. So vitamin D deficiency is associated with various reproduction problemes such as preeclampsia, insulin resistance, and gestational diabetes (5).

Research has shown that infertile women usually have lower levels of vitamin D (7). It was reported that, women who had ovulation defects were deficient in vitamin D, and that vitamin D can reduce inflammation in the body and help the fertility process (8). Another study showed that vitamin D levels in the follicular fluid had a significant positive effect on implantation and the pregnancy rate (9).

Previously it was shown that despite the positive effects of vitamin D as a hormone in the human body, its follicular fluid level did not have a significant effect on in vitro fertilization (IVF) outcomes (10). It was found that pregnancy rates were not associated with vitamin D levels in the follicular fluid. They declared that women who have higher levels of vitamin D have a significant decrease in fertilization rate, while increasing the implantation rate (11).

Glucose in the follicular fluid is one of the most important substances supporting oocyte metabolism (12). Glucose is the main energy source for all follicullar cells including oocytes and other somatic cells (13). Glucose was one of the regulatory factors for the GH-IGF-1 pathway in a study on evaluting vitamin D and glucose levels in the follicular fluid of 101 women who underwent IVF; it was found that there were insufficient vitamin D levels in the follicular fluid of the infertile women. Also, the vitamin D levels in the follicular fluid had a significant negative association with embryo quality. They reported that the follicular fluid glucose level was higher when vitamin D levels were insufficient (14).

Therefore, it is necessary to study the hormonal and biochemical changes of follicular fluid. Studies have shown different results about the effect of vitamin D and glucose in the follicular fluid on fertility and assisted reproductive technique (ART) outcomes. The aim of this study was to measure the levels of vitamin D and glucose in the follicular fluid and serum of infertile wemen who underwent IVF and evaluate the effects of vitamin D and glucose on ART outcomes.

## 2. Material and Methods

In this cross-sectional study, 102 infertile women (aged 20-40 yr) who were candidates for IVF/intracytoplasmic sperm injection (ICSI) who were referred to Afzalipour hospital of Kerman, Iran between March 2019 and February 2020 were enrolled.

Women were excluded if they had ovarian reserve depletion (defined as ≤ three oocytes with a conventional stimulation protocol, an antral follicular count of 5-7 follicles, Anti-Mullerian hormone levels of 0.5-1.1 ng/ml, or two episodes of polycystic ovarian response after maximal stimulation), reproductive anatomical defects, endocrine disorders, a history of kidney, liver or heart defects, vitamin D metabolism problems and/or a partner with severe male factor infertility.

All women were on an antagonist protocol for controlled ovarian hyperstimulation. Gonadotropin Cinal F (Cinal F, CinnaGen, Iran) was administered from day two of menstruation. The first dosage of gonadotropin was measured based on the patients' age and body weight (150-450 IU/day). On day seven of the stimulation all patients were monitored. When the dominant follicle size was 12 mm, the antagonist (Cetrorelix, Merck-Serono, Germany) was started. When the follicle's size was ≥ 18 mm in transvaginal ultrasonography imaging, final oocyte maturation was triggered by an intramuscular injection of 10,000 IU of human chorionic gonadotropin (pdpreg, Pooyesh Darou, Iran). After 34-36 hr, oocyte pickup was performed with vaginal ultrasound and general anesthesia. On the oocyte retrieval day, a sample was taken of peripheral blood for measuring vitamin D and fasting blood sugar (FBS). Then, follicular fluid containing oocytes was drained under a transvaginal ultrasound guide with general anesthesia. In the embryology lab, all oocytes were separated and the follicular fluid was transferred to the medical laboratory for determination of vitamin D and sugar level. The method for sample preparation was, first of all, centrifugation for 15 min at 1000 g and the seperated serum was kept at -20 C. For measurement of plasma and fulicular fluid vitamin D, enzyme immunoassay (Immunodiagnostic Ltd, UK) was used. Serum and folicular fluid glucose levels were estimated with oxidase methods by using a spectrophotometer system (Olympus Life and Material Science Europa, Hamburg, Germany). Serum glucose levels were defined as normal at < 110 ng/ml and pre-diabetic at ≥ 110 ng/ml.

The oocytes were fertilized in the embryology lab by ICSI and the number of fertilized oocytes was calculated two days after IVF/ICSI. Three days after oocyte retrieval, one or two high-quality embryos were transferred to the uterus. Implantation, chemical and clinical pregnancy rates, and abortion rates were assessed, and then the relationship between the serum and follicular fluid vitamin D and FBS levels, and the ART outcomes was assessed.

The implantation rate was measured based on the gestational sac in sonography per the number of embryos transferred. A positive chemical pregnancy was defined based on serum β-human chorionic gonadotropin two wk after embryo transfer. A positive clinical pregnancy was defined by at least one gestational sac in the uterine cavity under sonography 2-3 wk after the positive chemical pregnancy. The abortion rate was defined as pregnancy losses earlier than 20 wk of gestation per positive chemical pregnancies.

### Ethical considerations

This study was approved by the Ethical Committee of Kerman University of Medical Sciences, Kerman, Iran (Code: IR.KMU.REC.1397.513), and informed consent was obtained from all participants in this study.

### Statistical analysis

The Statistical Package for the Social Sciences (SPSS) software version 20 (IBM Co., Illinois, USA) was used for the data analysis. The normal distribution of the data was examined by the Kolmogorov-Smirnov test. Student's *t* test and proportional tests were performed for numerical variables and categorical variables, respectively. One-way ANOVA was used for assessing differences between the three groups of serum and follicular fluid vitamin D levels. The results were presented as mean ± standard deviation (SD) or frequency percentage (%). P-values less than 0.05 were considered statistically significant.

## 3. Results 

**Figure 1:**
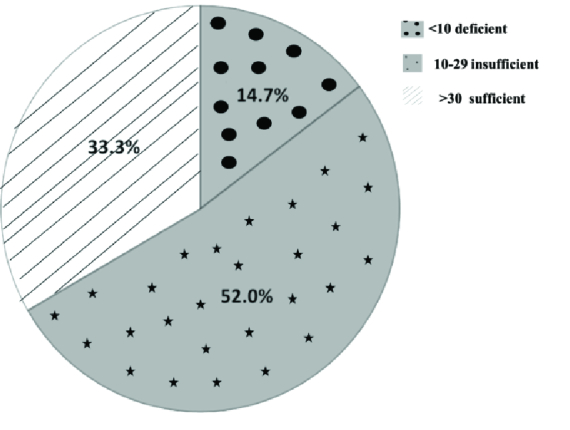
Prevalence of serum vitamin D status in the women.

Data for 102 women were analyzed in the present study. From these 102 women, in 18 cases clinical pregnancy was positive and in 84 cases clinical pregnancy was negative. Basic characteristics, serum vitamin D, follicular vitamin D, FBS, follicular fluid glucose and ART outcomes in the women with positive and negative clinical pregnancy are summarized in table I. Comparison between some parameters such as characteristics, serum vitamin D, follicular vitamin D, FBS and follicular fluid glucose in the two groups showed no significant difference. In terms of the ART outcomes, the numbers of matured oocytes and 2 pronucleus were significantly higher in the women with a positive clinical pregnancy (p = 0.01). But other parameters of ART outcomes were similar between the two groups (Table I). The serum vitamin D status of the cases showed that 66.7% of women were deficient or insufficient in vitamin D and only 33.3% had a sufficient status (Figure 1).

The comparison of glucose status and ART outcomes in women with deficient, insufficient, and sufficient vitamin D status is shown in table II. Based on vitamin D grouping, ART outcomes showed no significant difference. Follicular fluid glucose levels in women who were deficient, insufficient and sufficient in vitamin D were 65.20 ± 14.65, 63.47 ± 14.90 and 55.97 ± 15.64, respectively. Follicular fluid glucose levels were lower in women with sufficient vitamin D levels than in other groups and this difference was statistically significant (p = 0.01). In women with deficient serum vitamin D levels, the rates of chemical, clinical, and ongoing pregnancy were lower than in women with other vitamin D levels, but this difference was not statistically significant (Table II).

The assessment of study parameters based on follicular fluid vitamin D levels showed that there was no relationship between the three follicular fluid vitamin D levels and ART outcomes. In women who were deficient in follicular fluid vitamin D, the rates of chemical, clinical and ongoing pregnancy were lower than in other groups, but this was not statistically significant (Table III).

In table IV we summarized the serum and follicular fluid vitamin D levels and ART outcomes based on the FBS level. No significant difference was observed between the FBS levels and serum vitamin D or ART outcomes. The follicular fluid vitamin D level in women with normal and pre-diabetic FBS status was 29.34 ± 17.25 and 68.00 ± 31.11, respectively. In women with normal FBS levels, the level of follicular fluid vitamin D was significantly lower than in women with pre-diabetic FBS status (p < 0.01) (Table IV).

**Table 1:** Basic characteristics between the clinically pregnant and non-pregnant women


		**Positive clinical pregnancy (n = 18)**	**Negative clinical pregnancy (n = 84)**	**P-value**
Age (yr)*		28.83 ± 5.42	33.83 ± 5.063	0.61
BMI (kg/m^2^)*		25.33 ± 5.31	25.78 ± 5.15	0.57
Infertility duration (yr)*		5.80 ± 3.53	6.07 ± 3.80	0.78
Infertility type**				
	**Primary**	12 (66.7)	61 (72.6)	0.40
	**Secondary**	6 (33.3)	23 (27.4)	
Infertility etiology**				
	**Ovarian factor**	0 (0)	7 (8.3)	
	**Tubal factor**	4 (22.2)	19 (22.6)	
	**Male factor**	1 (5.6)	12 (14.3)	0.44
	**Mixed unknown**	5 (27.8)	13 (15.5)	
Gonadotropin dose*		2527.77 ± 1079.51	2975.89 ± 915.11		0.33
Stimulation days*		9.72 ± 2.58	10.77 ± 2.19		0.71
Serum vitamin D*		23.83 ± 12.57	25.53 ± 15.48	0.33
FF vitamin D*		28.83 ± 12.95	30.36 ± 19.16	0.09
Serum BS*		78.94 ± 10.16	82.14 ± 12.16	0.81
FF glucose*		58.66 ± 15.44	61.77 ± 15.47	0.85
Endometrial thickness*		9.41 ± 1.54	9.58 ± 1.74	0.37
Oocyte number*		11.55 ± 7.35	8.75 ± 5.70	0.08
MII number*		10.33 ± 6.99	7.34 ± 4.81	0.01
2PN number*		7.05 ± 5.91	4.75 ± 3.58	0.01
Embryo number*		5.66 ± 4.04	4.15 ± 3.14	0.25
Transferred embryos*		2.05 ± 0.53	1.91 ± 0.51	0.90
Embryo transfer quality**				
	**A**	7 (38.9)	15 (17.9)	0.25
	**B**	9 (50.0)	54 (64.2)	
	**C**	2 (11.1)	14 (16.7)	
	**D**	0 (0)	1 (1.2)	
*Data presented as Mean ± SD. **Data presented as n (%). Student's *t* test was used. A p-value below 0.05 was considered statistically significant. BMI: Body mass index, BS: Blood sugar, FF: Follicular fluid, 2PN: 2 pronuclear				

**Table 2:** Comparison of serum blood sugar and ART outcomes according to serum vitamin D level


	**Deficient vitamin D level (n = 15)**	**Insufficient vitamin D level (n = 53)**	**Sufficient vitamin D level (n = 34)**	**P-value**
Serum BS*	80.20 ± 12.66	82.16 ± 8.97	80.67 ± 13.15	0.75
FF glucose*	65.20 ± 14.65	63.47 ± 14.90	55.97 ± 15.64	0.01
Endometrial thickness*	9.40 ± 1.58	9.58 ± 1.82	9.57 ± 1.59	0.93
Oocyte number*	9.80 ± 5.68	9.15 ± 6.43	9.14 ± 5.85	0.93
MII number*	8.00 ± 4.44	8.01 ± 5.97	7.58 ± 4.76	0.93
2PN number*	5.13 ± 2.82	5.45 ± 4.76	4.70 ± 3.63	0.71
Embryo number*	3.66 ± 2.25	4.81 ± 3.92	4.14 ± 2.72	0.43
Fertilization**	77/120 (64.16)	289/425 (68.00)	160/258 (62.01)	0.90
Implantation**	3/32 (12.5)	13/102 (12.7)	10/64 (15.6)	0.97
Chemical pregnancy**	4 (16)	12 (48)	9 (36)	0.90
Clinical pregnancy**	3 (16.7)	9 (50.0)	6 (33.3)	0.96
Ongoing pregnancy**	2 (13.3)	8 (53.3)	5 (33.3)	0.98
Abortion**	2 (18.2)	4 (45.5)	4 (36.4)	0.89
*Data presented as Mean ± SD. **Data presented as n (%). One-way ANOVA was used. A p-value below 0.05 was considered statistically significant. BS: Blood sugar, FF: Follicular fluid, 2PN: 2 pronuclear				

**Table 3:** Comparison of blood sugar and ART outcomes according to follicular fluid vitamin D level


	**Deficient vitamin D level (n = 28)**	**Insufficient vitamin D level (n = 33)**	**Sufficient vitamin D level (n = 41)**	**P-value**
Serum BS*	81.10 ± 11.00	83.03 ± 10.88	81.04 ± 11.32	0.92
FF glucose*	64.85 ± 13.54	58.90 ± 15.47	60.60 ± 16.51	0.31
Endometrial thickness*	9.53 ± 1.48	9.53 ± 1.92	9.58 ± 1.67	0.98
Oocyte number*	7.89 ± 4.97	9.30 ± 6.76	10.12 ± 6.17	0.33
MII number*	6.71 ± 3.86	8.24 ± 6.32	8.36 ± 5.36	0.40
2PN number*	4.32 ± 2.61	5.54 ± 5.52	5.41 ± 3.71	0.45
Embryo number*	3.50 ± 2.11	4.78 ± 4.29	4.75 ± 3.12	0.23
Fertilization**	121/188 (64.16)	183/272 (68.00)	222/343 (62.01)	0.87
Implantation**	5/55 (9.09)	13/60 (21.66)	9/83 (10.84)	0.07
Chemical pregnancy**	5 (18.9%)	12 (36.4%)	8 (19.5%)	0.15
Clinical pregnancy**	4 (22.2%)	8 (44.4%)	6 (33.3%)	0.48
Ongoing pregnancy**	3 (20.0%)	7 (46.7%)	5 (33.3%)	0.44
Abortion**	2 (18.2%)	5 (54.5%)	3 (27.3%)	0.25
*Data presented as Mean ± SD. **Data presented as n (%). One-way ANOVA was used. A p-value below 0.05 was considered statistically significant. BS: Blood sugar, FF: Follicular fluid, 2PN: 2 pronuclear				

**Table 4:** Comparison of ART outcomes according to blood glucose level


	**Normal blood glucose level (n = 100)**	**Pre-diabetic blood glucose level (n = 2)**	**P-value**
Serum vitamin D*	24.87 ± 14.76	43.50 ± 19.09	0.08
FF vitamin D*	29.34 ± 17.25	68.00 ± 31.11	< 0.01
Endometrial thickness*	9.54 ± 1.71	10.00 ± 0.00	0.71
Oocyte number*	9.36 ± 6.08	3.50 ± 1.53	0.17
MII number*	7.97 ± 5.34	3.00 ± 2.82	0.19
2PN number*	5.20 ± 4.17	3.00 ± 2.82	0.46
Embryo number*	4.45 ± 3.36	3.00 ± 2.82	0.54
Fertilization**	520/797 (65)	6/6 (100)	0.06
Implantation**	26/195 (13.33)	1/3 (33.33)	0.54
Chemical pregnancy**	24 (96%)	1 (4%)	0.40
Clinical pregnancy**	18 (100%)	0 (0%)	0.51
Ongoing pregnancy**	15 (100%)	0 (0%)	0.55
Abortion**	9 (90.9%)	1 (9.1%)	0.07
*Data presented as Mean ± SD. **Data presented as n (%). Student's *t* test was used. A p-value below 0.05 was considered statistically significant. FF: Follicular fluid, 2PN: 2 pronuclear			

## 4. Discussion

Vitamin D is a main factor for the regulation of many biological activities in the human body. Vitamin D receptors have been found in the female reproductive system including in the ovary, uterus, endometrium and placenta after successful pregnancy (3). Vitamin D in the female reproductive system can regulate some of the cascades in the hypothalamic pituitary-gonadal axis and can affect the progress of oocyte maturation, endometrial growth and receptivity, implantation development, and progress of pregnancy (15). So, vitamin D may be an influential factor for successful ART cycles.

The aim of our study was to evaluate the role of serum and follicular vitamin D levels in influencing ART outcomes. We found that there was no relationship between the serum and follicular fluid vitamin D levels, and the outcomes of ART. Women with the lower level of vitamin D had lower rates of chemical, clinical and ongoing pregnancy than the women with sufficient levels of vitamin D, but this difference was not statistically significant. Similarly to our results, it was reported that there was no association between serum or follicular fluid vitamin D levels and the outcomes of ART (10, 16-18). In contrast to our findings, some researchers have reported that the women in their studies with vitamin D deficiency had lower rates of pregnancy than cases with sufficient vitamin D levels (7, 19-22).

The influence of serum and follicular fluid vitamin D has been considered for many yr. As we presented, some studies have suggested positive relationships between serum and follicular fluid vitamin D, and pregnancy rates, while some studies have not found such a relationship. This could be due to differences in the study design, case selection, infertility type, socioeconomic status, age, body mass index and/or laboratory tests.

It was reported that when vitamin D receptors were blocked in experimental animals, the oogenesis process was disrupted and fertility decreased (23). In another study it was demonstrated that in cases with serum vitamin D sufficiency the implantation rate was higher than in cases with serum vitamin D deficiency. They concluded that perhaps vitamin D is more influential in endometrial receptivity than the oogenesis process (20).

Two separate studies reported that in cases with vitamin D deficiency, some changes occurred in calcium-phosphate metabolism. This alteration can affect endometrial receptivity and ovary function (24, 25). In several experimental animal studies, researchers found that hypocalcemia following vitamin D deficiency was responsible for decreased pregnancy rates and fertility (26, 27). Some studies have shown that calcium and vitamin D metabolism can induce aromatase gene expression (28, 29). Aromatase in the ovary and uterus induces molecular cascades leading to successful ovulation, endometrial growth, and implantation (30). So, alterations in normal vitamin D levels can change the calcium and aromatase sequence. This is an important factor for changes in fertility, ovulation, embryo formation, implantation, placenta formation, and pregnancy progression. Based on the above studies, serum vitamin D can play an important role in the female reproductive system, but the molecular process or main effective points are still unknown.

The results of the present study showed that in the serum vitamin D sufficient group, the level of follicular fluid glucose was significantly lower than in the deficient and insufficient groups. Anifandis and colleagues evaluated serum vitamin D in combination with glucose and their role in IVF outcomes. They reported that follicular fluid glucose was higher when vitamin D was insufficient (14), which is in accordance with our results.

Some studies have reported the role of vitamin D in inducing insulin secretion and controlling glucose metabolism. These have shown that vitamin D elevates insulin secretion (31, 32). It is possible that normal vitamin D levels promote insulin secretion and then insulin increases glucose utilization by oocytes and other cells in the follicular cavity, including granulosa and cumulus cells. Consequently, this could be responsible for the lower levels of follicular sugar in cases with sufficient vitamin D levels.

Also, in the present study, we assessed the FBS and follicular glucose and calculated the correlation between FBS and ART outcomes. The results showed that there was no correlation between FBS and follicular glucose, and ART outcomes. The relationship between FBS and follicular vitamin D was statistically significant. Several studies have focused on the role of blood sugar in the reproductive system. In cases with high levels of FBS and in diabetic cases, the rate of fertility is higher than in normal blood sugar cases (33, 34). Researchers have discussed that high sugar levels and low insulin levels may be responsible for defects in fertility and decreases in pregnancy rates (35, 36). There is no evidence demonstrating the relationship between FBS and follicular vitamin D. We suggest that the lower levels of follicular fluid vitamin D in cases with normal FBS may be due to sugar metabolism. Perhaps secreted insulin in cases with normal FBS can affect vitamin D metabolism in the follicular fluid. However, this theory needs more experimental and clinical studies to confirm the validity.

## 5. Conclusion

The results of this study demonstrated some evaluated parameters including serum vitamin D levels, follicular fluid vitamin D levels, FBS and follicular fluid sugar did not have any effect on ART outcomes. However, in women with lower levels of vitamin D in the blood and follicular fluid, the rate of pregnancy was lower than in the vitamin D sufficient group. We recommend that future studies have larger sample sizes and consider different infertility etiologies or vitamin D supplementation in infertile women to further understand the relationship between vitamin D, FBS or follicular fluid sugar and ART outcomes.

##  Conflict of Interest

The authors declare that there is no conflict of interest.
